# Multilevel Factors Affecting Healthcare Workers’ Perceived Stress and Risk of Infection During COVID-19 Pandemic

**DOI:** 10.3389/ijph.2021.599408

**Published:** 2021-03-05

**Authors:** Gilbert T. Chua, Keith T. S. Tung, Mike Yat Wah Kwan, Rosa S. Wong, Celine S. L. Chui, Xue Li, Wilfred H. S. Wong, Winnie W. Y. Tso, King Wa Fu, Ko Ling Chan, Yun Kwok Wing, Eric Yu Hai Chen, Tatia Mei Chun Lee, Nirmala Rao, Godfrey C. F. Chan, Ellis K. L. Hon, Ivan Fan Ngai Hung, Kui Kai Lau, Marco H. K. Ho, Kirstie Wong, Xiaoli Xiong, Shuiqing Chi, Shao-tao Tang, Paul K. H. Tam, Ian C. K. Wong, Patrick Ip

**Affiliations:** ^1^ Department of Paediatrics and Adolescent Medicine, LKS Faculty of Medicine, The University of Hong Kong, Hong Kong SAR, China; ^2^ Department of Paediatrics and Adolescent Medicine, Princess Margaret Hospital, Hong Kong SAR, China; ^3^ Centre for Safe Medication Practice and Research, Department of Pharmacology and Pharmacy, The University of Hong Kong, Hong Kong SAR, China; ^4^ Department of Medicine, LKS Faculty of Medicine, The University of Hong Kong, Hong Kong SAR, China; ^5^ Journalism and Media Studies Centre, The University of Hong Kong, Hong Kong SAR, China; ^6^ Department of Applied Social Sciences, The Hong Kong Polytechnic University, Hong Kong SAR, China; ^7^ Department of Psychiatry, Faculty of Medicine, The Chinese University of Hong Kong, Hong Kong SAR, China; ^8^ Department of Psychiatry, LKS Faculty of Medicine, The University of Hong Kong, Hong Kong SAR, China; ^9^ State Key Laboratory of Brain and Cognitive Science, The University of Hong Kong, Hong Kong SAR, China; ^10^ Faculty of Education, The University of Hong Kong, Hong Kong SAR, China; ^11^ The Hong Kong Children’s Hospital, Hong Kong SAR, China; ^12^ Department of Integrated Chinese and Western Medicine, Wuhan Children’s Hospital (Wuhan Maternal and Child Healthcare Hospital), Tongji Medical College, Huazhong University of Science and Technology, Wuhan, China; ^13^ Department of Paediatric Surgery, Union Hospital, Tongji Medical College, Huazhong University of Science and Technology, Wuhan, China; ^14^ Department of Surgery, LKS Faculty of Medicine, The University of Hong Kong, Hong Kong SAR, China; ^15^ Dr. Li Dak Sum Research Centre, The University of Hong Kong-Karolinska Institutet Collaboration in Regenerative Medicine, The University of Hong Kong, Hong Kong SAR, China; ^16^ Research Department of Practice and Policy, UCL School of Pharmacy, University College London, London, United Kingdom

**Keywords:** COVID-19, healthcare workers, perceived stress, psychological wellbeing, hospital policies

## Abstract

**Objectives:** This study aimed to identify key factors affecting Healthcare workers (HCWs) perceived stress and risk of contracting COVID-19 among themselves and their family members during the pandemic.

**Methods:** A cross-sectional online questionnaire study was conducted between 19 March and April 5, 2020 in Hong Kong. HCWs from public hospitals and private dentists, and their family members participated.

**Results:** A total of 747 HCWs and 245 family members participated. Higher perceived stress in HCWs was associated with more negative changes in family relationship (*p* = 0.025). The HCWs’ perceived stress, however, was positively associated with family cohesion (*p* = 0.033) and stress levels of family members (*p* < 0.001). The level of HCWs’ satisfaction toward the hospital policies in response to the COVID-19 outbreak was associated with lower levels of perceived stress and risk of themselves or their family members contracting COVID-19. HCWs’ previous frontline experience of SARS was significantly associated with less perceived risk of themselves or their family members contracting COVID-19.

**Conclusion:** Hospital policies addressing HCWs’ needs, frontline experience of SARS, and family relationship influenced psychological wellbeing of HCWs during the COVID-19 outbreak.

## Introduction

The coronavirus disease 2019 (COVID-19) pandemic due to severe acute respiratory syndrome coronavirus 2 (SARS-CoV-2) has already caused more than 34 million confirmed cases and over one million deaths worldwide as of early-October 2020 [1]. Healthcare workers (HCWs) have a very high risk of being infected by SARS-CoV-2 and are increasingly worried about potentially infecting their family members [2, 3]. This situation is worsen by the chronic shortage of personal protective equipment (PPE) worldwide [4]. Recent reports have revealed that exposure to SARS-CoV-2 while caring for patients due to lack of PPE has led to many HCWs becoming infected by COVID-19 and many have sadly died. The unfamiliarity and perceived uncontrollability of the COVID-19 might hamper the psychological wellbeing of the HCWs.

Previous evidence already suggest that HCWs are susceptible to psychological distress, anxiety, post-traumatic stress disorder, sleep disturbances, and burnout during and after major infectious disease outbreaks [5–7]. Risk factors affecting their wellbeing include beliefs about their increased likelihood of infection, heightened concern of infecting their family members, increased workload, stress, lack of available precautionary measures, nature of their work, and family structures [8–10]. During the SARS outbreak, HCWs in Hong Kong who survived from SARS infection were 2.24 times more likely to develop psychiatric morbidities than non-HCW survivors, which lasted up to 3.5 years after the end of the epidemic [11]. In the COVID-19 pandemic, HCWs in China reported high levels of anxiety, and stress, and low self-efficacy, with associated poor sleep quality and weak social support [12]. However, only a few studies focused on the risk and protective factors contributing to HCWs’ psychological wellbeing. Understanding the contributing factors to HCWs’ psychological wellbeing would provide insightful advice on how to mitigate the escalating stress level of HCWs during infectious diseases outbreak. An investigation of the psychological wellbeing of HCWs during the COVID-19 outbreak and the factors affecting their mental health is urgently warranted.

This study aimed to examine the levels of HCWs’ perceived stress and risk of infection by COVID-19 while caring for patients during the pandemic and to identify how individual, family, and policy factors, and previous frontline experience during the SARS 2003 outbreak influenced these outcomes.

## Methods

### Study Participants

This was a cross-sectional online questionnaire study of HCWs and their family members at the peak of the COVID-19 pandemic in Hong Kong. The inclusion criteria for participation in the study included HCWs working in the Hong Kong public healthcare system such as doctors, dentists, nurses, allied health professionals, pharmacists, and healthcare assistants; dentists working in the private sector; and both HCWs and their family members aged 18 years or above. We disseminated the online survey through the public hospital network and the web-link was published on several websites affiliated with key HCW associations in Hong Kong. After providing consent electronically, recruited subjects (HCWs and their family members) were invited to complete the questionnaire, which was available in both English (Supplementary Material) and Chinese. We linked the responses of HCWs and their family members using a unique identification number.

### Measurements

The study questionnaires covered basic characteristics, mental health status, family relationships, and risk perception of HCWs and family members contracting COVID-19.

#### Demographics

We obtained detailed information from HCWs regarding the nature of their job, experience of managing suspected or confirmed COVID-19 cases and cohabitation status, as well as previous frontline experience of the SARS 2003 outbreak.

#### Perceived Stress

Both HCWs and their family members were asked to rate their perceived stress level respectively using the 10-item Perceived Stress Scale (PSS-10), which is a widely used instrument for measuring psychological distress that has been validated in the Chinese population [13]. The 10 items are scored on a 5-point scale from 0 to 4 and the total score is out of 40. A higher score indicated higher level of perceived stress.

#### Perceived Family Cohesion and Changes in Family Relationships

Both HCWs and their family members were asked to rate separately their perceived family support using the Family APGAR (Adaption, Partnership, Growth, Affection, Resolve) scale, which has been validated in the Chinese population [14]. This scale has five items rated on a 3-point scale and a total score out of 15. A higher total score indicated better perceived family cohesion. They were also asked to rate the perceived change in family relationship during the COVID-19 outbreak.

#### Risk Perception

The HCWs were asked “How bad do you feel if you were diagnosed with COVID-19” and to rate their perceived risk of infection by COVID-19 on a 5-point scale from “not bad at all” to “very bad.” This reflected how badly they would feel if they or their family members were diagnosed with COVID-19. A higher score indicated a higher perceived risk of infection.

#### Satisfaction Toward Hospital Policies in Response to the COVID-19 Outbreak

The HCWs were asked to rate their satisfaction toward hospital policies in response to the COVID-19 outbreak, which included the provision of PPE for those working in high-risk areas (e.g., isolation wards), PPE for those working in non-high-risk areas (e.g., outpatient clinics), special accommodation allowance for HCWs working in high-risk areas to stay at hotels to prevent transmitting COVID-19 to family members, and special salary increments for HCWs working in high-risk areas. The four items were rated on a 5-point scale between −2 and +2, and the total score represented the overall satisfaction. As most dentists in Hong Kong work in the private sector, their responses were excluded from this analysis.

### Data Analysis

We used Student’s *t*-test, Chi-square test and Fisher’s exact test to compare the perceived stress and risk of being diagnosed with COVID-19 across different groups as appropriate. The relationships between the study variables were examined using bivariate correlations. A series of regression models were conducted to explore the factors associated with HCWs’ perceived levels of stress and risk of infection during the COVID-19 outbreak. Univariate regression analyses were first conducted to measure the crude independent effects of different factors including age, role in healthcare sector, perceived level of family cohesion and changes in family relationships during COVID-19 outbreak, satisfaction toward the overall and individual hospital special policies, and measures of HCWs, and perceived stress levels and family relationships of family members. Multiple regression models were performed to examine the adjusted associations while controlling for the impacts of sex, marital status, role in healthcare sector, age, and number of years of work experience. All statistical analyses were conducted using SPSS version 25.0 (IBM, Armonk, New York, United States). All statistical tests were two-tailed and a *p*-value of less than 0.05 was considered statistically significant.

## Result

Between 19 March and April 5, 2020, a total of 747 HCWs and 245 family members completed the online questionnaires. Table 1 summarizes the demographics of HCWs who participated in this study and the overall responses. The mean age of HCWs was 39.6 years (SD = 10.9), 27% were male, and over 60% were married or cohabitating. The average years of work experience was 13 years (SD = 7.7). The majority of HCWs were nurses (45.7%) and doctors (23.0%). Approximately 20% (149/747) of the respondents worked in COVID-19 high-risk areas and 10.4% (78/747) had direct contact with confirmed COVID-19 patients. Over 90% of HCWs experienced the SARS outbreak in Hong Kong, with 44.2% working in the healthcare sector during the SARS outbreak. About 15% of HCWs reported they had a peer, colleague, or family member that had been infected or died due to SARS. The mean stress levels of HCWs and their family members were 21.3 and 21.1, respectively. Family cohesion scores of HCWs and their family members were 11.8 and 12.0 respectively. Family relationships during the COVID-19 outbreak were reported to be improved in 23.0% of HCWs, but deteriorated in 10.04%, whereas 66.8% reported to have no changes. Overall, HCWs reported dissatisfaction of hospital support in response to the COVID-19 outbreak.

**TABLE 1:** Healthcare worker demographics and overall responses in Hong Kong between 19 March and April 5, 2020.

Healthcare worker characteristics	N (%)	Mean (SD)
Age (years)	—	39.59 (10.87)
Work experience in the healthcare sector (years)	—	13.00 (7.73)
Sex		
Male	202 (27.04)	—
Female	544 (72.82)	—
Marital status		
Single	273 (36.55)	—
Married/Cohabitating	474 (63.45%)	—
Roles		
Doctors	172 (23.03)	—
Dentists	33 (4.42)	—
Nurses	341 (45.65)	—
Pharmacists	20 (2.68)	—
Allied health professionals	82 (10.98)	—
Healthcare assistants/Others	99 (13.25)	—
Working in high-risk area	149 (19.95)	—
Living separately from family during the COVID-19 outbreak	74 (9.91)	—
Had direct contact with COVID-19 patients	78 (10.44)	—
Experienced SARS outbreak in Hong Kong	690 (92.37)	—
Working in healthcare sector during the SARS outbreak	330 (44.18)	—
Being frontline worker during the SARS outbreak	127 (17.00)	—
Had peers/colleagues/relatives infected or died due to SARS	111 (14.86)	—
HCWs’ perceived level of stress (Range 0–40)	—	21.32 (3.38)
HCWs’ perceived family cohesion (Range 5–15)	—	11.75 (2.78)
HCWs’ perceived change in family relationships		
Much worse	5 (0.67)	—
Worse	70 (9.37)	—
Same	499 (66.80)	—
Better	143 (19.14)	—
Much better	29 (3.88)	—
HCWs’ perceived risks of COVID-19 infection (Range 1–5)	—	4.07 (0.90)
HCWs’ perceived FM risk of COVID-19 infection (Range 1–5)	—	4.49 (0.77)
HCWs’ overall satisfaction with hospital support (Range −2 – +2)	—	−0.58 (0.93)
Satisfaction with PPE provision in high-risk areas (Range −2 – +2)	—	−0.73 (1.08)
Satisfaction with PPE provision in non-high-risk areas (Range −2 – +2)	—	−0.75 (1.06)
Satisfaction with the provision of accommodation allowance (Range −2 – +2)	—	−0.42 (1.09)
Satisfaction with the provision of special salary increments (Range −2 – +2)	—	−0.40 (1.09)
FMs’ perceived level of stress (Range 0–40)	—	21.10 (3.38)
FMs’ perceived family cohesion (Range 5–15)	—	11.96 (2.64)

COVID-19, coronavirus disease 2019; FMs, family members; HCWs, healthcare workers; PPE, personal protective equipment; SARS, severe acute respiratory syndrome; SD, standard deviation.

Table 2 describes mean levels of perceived stress and risk of COVID-19 infection of HCWs and their family members by participants’ characteristics. Between-group comparisons showed that HCWs in different roles had significantly different mean stress scores and mean risk scores for COVID-19 infection for themselves and their family members (all *p* < 0.05). The HCWs who worked in the healthcare sector and those on the frontline caring for SARS patients had significantly lower perceived risk of COVID-19 infection for themselves and their family members (all *p* < 0.05). However, HCWs working in high-risk areas and their decision to live separately from their families was not correlated with their perceived levels of stress and risk of COVID-19 infection.

**TABLE 2:** Healthcare workers’ perceived levels of stress and risk of contracting COVID-19 in Hong Kong between 19 March and April 5, 2020.

Healthcare worker characteristics	HCWs’ mean perceived level of stress (SD) [Range 0–40]	*p*	HCWs’ perceived risk of COVID-19 infection (SD) [Range 1–5]	*p*	HCWs’ perceived FM risk of COVID-19 infection (SD) [Range 1–5]	*p*
Sex		0.963		0.355		0.633
Male	21.31 (3.38)		4.11 (0.89)		4.47 (0.77)	
Female	21.32 (3.38)		4.05 (0.90)		4.50 (0.77)	
Marital status		0.835		0.991		0.026
Single	21.28 (3.37)		4.07 (0.90)		4.57 (0.66)	
Married/Cohabitating	21.34 (3.38)		4.07 (0.90)		4.44 (0.83)	
Role		<0.001		0.007		0.003
Doctors	21.59 (3.16)		4.03 (0.86)		4.52 (0.63)	
Dentists	21.88 (2.62)		4.52 (0.71)		4.76 (0.61)	
Nurses	21.57 (3.10)		4.09 (0.85)		4.50 (0.75)	
Pharmacists	22.20 (2.55)		3.90 (1.02)		4.55 (0.60)	
Allied health professionals	20.87 (4.41)		4.18 (0.82)		4.61 (0.73)	
Healthcare assistants/Others	19.95 (3.73)		3.83 (1.12)		4.21 (1.07)	
Working in high-risk area		0.159		0.159		0.713
Yes	21.66 (3.45)		3.97 (1.01)		4.47 (0.77)	
No	21.23 (3.36)		4.09 (0.86)		4.50 (0.77)	
Living separately from family during the COVID-19 outbreak		0.131		0.079		0.669
Yes	21.88 (3.54)		3.89 (1.01)		4.53 (0.71)	
No	21.25 (3.36)		4.08 (0.88)		4.49 (0.78)	
Direct contact with COVID-19 confirmed cases		0.143		0.007		0.726
Yes	21.85 (3.18)		3.81 (1.01)		4.46 (0.77)	
No	21.25 (3.40)		4.10 (0.88)		4.49 (0.77)	
Experienced SARS outbreak in Hong Kong		0.072		0.673		0.726
Yes	21.38 (3.17)		4.07 (0.88)		4.49 (0.76)	
No	20.54 (5.26)		4.01 (1.04)		4.46 (0.89)	
Working in healthcare sector during the SARS outbreak		0.462		0.001		<0.001
Yes	21.42 (3.26)		3.95 (0.88)		4.36 (0.83)	
No	21.24 (3.46)		4.16 (0.90)		4.59 (0.71)	
Being frontline worker during the SARS outbreak		0.642		0.015		0.001
Yes	21.19 (3.45)		3.89 (0.89)		4.29 (0.86)	
No	21.34 (3.36)		4.10 (0.89)		4.53 (0.75)	
Had peers/colleagues/relatives infected or died due to SARS		0.926		0.793		0.553
Yes	21.29 (3.81)		4.05 (0.84)		4.45 (0.76)	
No	21.32 (3.30)		4.07 (0.91)		4.50 (0.77)	

COVID-19, coronavirus disease 2019; FMs, family members; HCWs, healthcare workers; PPE, personal protective equipment; SARS, severe acute respiratory syndrome; SD, standard deviation.

Correlation analyses (Table 3) demonstrated the HCWs’ perceived stress was positively associated with their perceived risk of COVID-19 infection (r = 0.218), family members’ risk of COVID-19 infection (r = 0.199), and family members’ stress levels (r = 0.237) (all *p* < 0.001). Whereas, both HCWs’ perceived risk of COVID-19 infection for themselves and their family members were negatively correlated with their satisfaction with hospital policies (r = −0.219–−0.278, *p* < 0.001). Age and years of work experience were also negatively correlated with their perceived risk of COVID-19 infection for themselves and their family members.

**TABLE 3:** Pearson correlation matrix for continuous variables between the characteristics of healthcare workers in Hong Kong between 19 March and April 5, 2020.

	Healthcare worker characteristics	1	2	3	4	5	6	7	8	9	10	11	12	13	14
1	Age	—	0.763***	0.471***	−0.012	0.028	−0.159***	−0.218***	0.383***	0.334***	0.034***	0.327***	0.318***	0.025	−0.036
2	Years of working in the healthcare sector		—	0.508***	0.001	0.072*	−0.112**	−0.169***	0.324***	0.295***	0.293***	0.258***	0.269***	−0.033	0.017
3	Number of children			—	−0.033	0.136***	−0.022	−0.009	0.112**	0.013***	0.139***	0.064	0.053	0.095	−0.002
4	HCWs’ perceived level of stress				—	0.079*	0.218***	0.199***	−0.054	−0.015	−0.053	−0.057	−0.061	0.237***	0.094
5	HCWs’ perceived family cohesion					—	−0.009	−0.042	0.071	0.095**	0.074*	0.041	0.035	0.018	0.328***
6	HCWs’ perceived risk of COVID-19 infection						—	0.648***	−0.278***	−0.219***	−0.224***	−0.263***	−0.249***	0.066	−0.116
7	HCWs’ perceived FM risk of COVID-19 infection							—	−0.201***	−0.175***	−0.168***	−0.018***	−0.017***	0.083	−0.079
8	Overall satisfaction with special support policies								—	0.851***	0.834***	0.883***	0.875***	−0.026	0.008
9	Satisfaction with the provision of PPE in high-risk areas									—	0.765***	0.589***	0.058***	−0.006	0.003
10	Satisfaction with the provision of PPE in non-high-risk areas										—	0.566***	0.055***	−0.025	0.085
11	Satisfaction with the provision of accommodation allowance											—	0.877***	−0.001	0.094
12	Satisfaction with the provision of special salary increments												—	−0.008	0.098
13	FMs’ perceived level of stress													—	0.073
14	FMs’ perceived family cohesion														—

*p < 0.05, **p < 0.01, ***p < 0.001.

COVID-19, coronavirus disease 2019; FMs, family members; HCWs, healthcare workers; PPE, personal protective equipment.

Table 4 displays the adjusted association of different factors with the HCWs’ perceived levels of stress and risk of COVID-19 infection for themselves and their family members. Doctors nurses and dentists were found to have significantly higher level of stress compared to allied health professionals, healthcare assistants, and others (*p* = 0.001). Higher perceived stress in HCWs was associated with more negative changes in family relationship (*p* = 0.025). The HCWs’ perceived stress level was also positively associated with their perceived family cohesion (*p* = 0.033), and stress levels of family members (*p* < 0.001). The HCWs’ satisfaction with overall and individual special hospital policies in response to the COVID-19 outbreak were negatively associated with their perceived levels of stress (all *p* < 0.05, except PPE provision in high-risk areas), and risk of contracting COVID-19 for themselves and their family members (all *p* < 0.001). HCWs with experience of working in healthcare sector or with frontline experience during SARS outbreak were found to have significantly lower perceived risk of COVID-19 infection for themselves and their family members (all *p* < 0.01).

**TABLE 4:** Adjusted associations between individual, family, and policy factors with outcomes of healthcare workers in Hong Kong between 19 March and April 5, 2020.

	HCWs’ perceived level of stress		HCWs’ perceived risk of COVID-19 infection		HCWs’ perceived FM risk of COVID-19 infection	
	β (95% CI)	*p*	β (95% CI)	*p*	β (95% CI)	*p*
Age^a^	0.003 (−0.01, 0.01)	0.591	−0.02 (−0.03, −0.004)	0.006	−0.02 (−0.03, −0.01)	0.002
Role						
Doctors/dentists/nurses^a^	0.29 (0.12, 0.46)	0.001	0.09 (−0.07, 0.26)	0.267	0.10 (−0.06, 0.27)	0.212
Allied health professionals/healthcare assistants/others	Ref	—	Ref	—	Ref	—
Change in family relationships during the COVID-19 outbreak^a^	−0.12 (−0.23, −0.02)	0.025	−0.09 (−0.20, 0.02)	0.120	−0.11 (−0.22, −0.01)	0.038
Perceived family cohesion^a^	0.03 (0.002, 0.05)	0.033	−0.01 (−0.04, 0.02)	0.543	−0.02 (−0.05, 0.01)	0.159
Overall satisfaction with special support policies^a^	−0.10 (−0.19, −0.02)	0.020	−0.31 (−0.39, −0.23)	<0.001	−0.19 (−0.27, −0.10)	<0.001
Satisfaction with the provision of PPE in high-risk areas^a^	−0.04 (−0.11, 0.03)	0.258	−0.21 (−0.28, −0.14)	<0.001	−0.13 (−0.21, −0.06)	<0.001
Satisfaction with the provision of PPE in non-high-risk areas^a^	−0.08 (−0.16, −0.01)	0.028	−0.21 (−0.29, −0.14)	<0.001	−0.13 (−0.20, −0.05)	<0.001
Satisfaction with the provision of accommodation allowance^a^	−0.08 (−0.15, 0.00)	0.036	−0.23 (−0.30, −0.16)	<0.001	−0.13 (−0.20, −0.06)	<0.001
Satisfaction with the provision of salary increments^a^	−0.09 (−0.16, −0.02)	0.017	−0.22 (−0.29, −0.15)	<0.001	−0.13 (−0.20, −0.06)	<0.001
Working in the healthcare sector during the SARS outbreak^b^	0.01 (−0.14, 0.17)	0.879	−0.27 (−0.43, −0.12)	<0.001	−0.31 (−0.46, −0.15)	<0.001
Frontline experience during the SARS outbreak^b^	−0.10 (−0.29, 0.09)	0.314	−0.26 (−0.46, −0.07)	0.009	−0.31 (−0.51, −0.12)	0.002
Peers/colleagues/relatives infected or died due to SARS^b^	−0.07 (−0.27, 0.13)	0.496	−0.05 (−0.25, 0.16)	0.656	−0.07 (−0.27, 0.14)	0.512
FMs’ perceived level of stress^a^	0.07 (0.03, 0.10)	<0.001	0.02 (−0.02, 0.06)	0.399	0.02 (−0.02, 0.05)	0.387
FMs’ perceived family cohesion^a^	0.03 (−0.01, 0.08)	0.140	−0.05 (−0.11, −0.002)	0.044	−0.03 (−0.08, 0.01)	0.163

^a^ Model adjusted for sex, marital status, role of the healthcare worker, age, and number of years working in healthcare sector.

^b^ Age and number of years working in healthcare sector were not included.

COVID-19, coronavirus disease 2019; FMs, family members; HCWs, healthcare workers; PPE, personal protective equipment; SARS, severe acute respiratory syndrome; SD, standard deviation.

## Discussion

This study was conducted at the peak of the COVID-19 outbreak in Hong Kong [15]. To the best of our knowledge, this study is one of the first to examine HCWs’ perceived levels of stress and their perceived risk of contracting COVID-19 for themselves and their family members when the government is undertaking a set of measures to protect citizens and communities. This is also the first study to examine how previous frontline experience of the SARS outbreak and family factors are associated with HCWs’ psychological wellbeing during the pandemic.

The SARS epidemic in Hong Kong is unique as not all regions of the world were severely affected by the SARS outbreak. Our study showed that the experience as frontline workers during the SARS outbreak was associated with a lower perceived risk of contracting COVID-19 for the HCWs and their family members after adjusting for sex, marital status, and role. Age and years of experience were not included in the adjusted model, as all HCWs who had experienced the SARS epidemic were senior staff. Such observation is possibly explained by the fact that SARS-CoV and SARS-CoV-2 shared similarities in terms of their genomic sequences and biology [16]. This may also signifies that experience in the COVID-19 pandemic could benefit HCWs in facing infectious disease outbreaks in the future.

The mean stress scores in this study indicated a moderate level of stress among the HCWs and their family members in fighting COVID-19, which was similar to other parts of the world, including China, Singapore, North America, the Middle East and Europe [17–21]. The perceived levels of stress in HCWs were significantly and negatively associated with changes in family relationships over the COVID-19 outbreak period. Family support has been proposed to mitigate psychological distress experienced by HCWs particularly in the face of the overwhelming burden of COVID-19 onto the healthcare system [22]. Another stressor for HCWs was the stress and anxiety experienced by their family members during the outbreak. It is inevitable that laypersons would also suffer from mental health disturbances in this pandemic. This is not only because of the fear of contracting the diseases but also due to a dramatic change in lifestyles, social and political atmospheres and economical impact [23, 24]. It is possible that the vicious circle of stressful responses between HCWs and their family members might worsen the health and wellbeing of each other. We also observed a positive correlation between HCWs’ perceived risk of contracting COVID-19 for themselves and their family members, suggesting the possibility of higher apprehension in the HCWs due to the perception of their own infection risk and also their family members’ risk of getting infected. In addition, concerns about the health of the family and fear of spreading the infection to family members can be other sources of stress [9, 25, 26]. In Canada and the United States, many HCWs have opted to live separately from their family, such as paying to stay at a hotel or in donated recreational vehicles [27, 28]. Our study demonstrated that satisfaction toward allowances for accommodation could considerably alleviate HCWs’ stress and their perceived risk of infecting family members. The Hong Kong Hospital Authority implemented a special salary increment and rental allowance scheme, and liaized with local hotels to support HCWs to isolate themselves from their families while working in high-risk areas [29, 30]. We found that 55.4% of HCWs who had been in direct contact with COVID-19 patients also opted to live away from their family during the COVID-19 outbreak, indicating that accommodation support is needed in a pandemic. This concurs with a recent study in Saudi Arabia that living with co-habitants predicted higher level of concerns among HCWs [21].

Our study demonstrated that HCWs’ perceived level of stress was significantly associated with their job role. Doctors, nurses, and dentists who were frequently in contact with infected patients experienced higher levels of stress than other supporting staff. Standard precautions including barrier protection, such as masks, face-shields, and disposal gowns, are recommended to protect them from airborne infections, and was proven to be effective in Hong Kong where no HCWs were being infected with COVID-19 in their workplace [31–33]. This was supported by our study results that satisfaction with hospital’s provision of PPE can significantly reduce HCWs’ perceived level of stress and risk of contracting COVID-19 for themselves and their family members. However, the global shortage of PPE and the lack of government support may have aggravated the perceived risk of infection of HCWs during the COVID-19 outbreak. It has been reported that some HCWs threatened to stop working due to the lack of PPE [34]. These reports further highlight the profound impacts of worldwide PPE shortage during the COVID-19 pandemic on the psychological wellbeing of HCWs. To overcome the challenges, the Hong Kong government has implemented the policy of stockpiling PPE to protect the health and wellbeing of our frontline healthcare workers during the infectious disease outbreaks. During the SARS outbreak, a high number of HCWs who worked in intensive care units were infected from exposure to high-risk procedures, including the use of nebulizers, endotracheal suction, and intubation [35, 36]. These SARS experiences have led the Hong Kong government to regularly update and improve guidelines for frontline staff in the triage of suspected cases and in implementing protective measures for HCWs [37]. For example, after the SARS epidemic, ward facilities in Hong Kong underwent substantial improvements with modified protocols for those with suspected infectious diseases. Contingency plans were established to ensure 3-months stocks of PPE and the availability of portable high-efficiency particulate air filters in clinical areas where permanent installation is not feasible [38]. These special preventive measures adopted in Hong Kong might serve as a good example to alleviate the psychological impacts on HCWs during infectious diseases outbreak. Concurring with a recent study in Hong Kong surveying more than 1,000 non-HCW, it demonstrated that workplace infection control policies and employees’ perception of infection risk directly impact on employees’ health outcomes, which the latter partly mediated the relationship between workplace infection control policies their health outcomes [39].

Recent studies have now focused on intervention to alleviate the adverse psychological impact of the COVID-19 pandemic on HCWs. In the United States, a multidisciplinary peer support model known as “Battle Buddies” was developed for the management of psychological stress exposure in providers deployed to disasters [40]. In China, the National Health Commission implemented policies with the joint effort of psychiatrists and mental health experts to handle emergency psychological crisis as a result of the COVID-19 outbreak. This policy targets specifically six high risks populations, including HCW, confirmed patients, their close contacts and other susceptible persons in the general public [41]. Digital platforms were also utilized in the United Kingdom to provide evidence-based support HCWs and their peers and families related to their psychological wellbeing [42]. As new waves of COVID-19 outbreaks was being anticipated in the coming winter, ongoing research in monitoring as well as the provision of timely intervention to mitigate the psychosocial wellbeing of HCWs and their families around the world will be needed.

This study had several limitations. First, this cross-sectional survey was carried out in Hong Kong during an outbreak period and did not measure the HCWs’ levels of stress and mental health status during the pre-outbreak period as comparison baseline levels. Second, the level of psychological distress in HCWs may change and hence this cross-sectional survey may not capture the fluctuations in stress levels over the highly dynamic course of the pandemic. Nevertheless, the study was conducted in the peak period of the COVID-19 outbreak [15] which was considered as the most stressful period of the pandemic and thus it should involve minimal recall bias. Third, the survey was distributed through healthcare worker networks and associations, which might be prone to potential sampling and volunteer bias. Respondents of our survey represented 2.7% of all doctors and 1.2% of all nurses employed by the Hong Kong Hospital Authority. Our respondents involved HCWs working in both high-risk and non-high risks areas, with a wide spectrum of working experiences (mean 13 years, SD 7.73). The mean age of our respondents was approximately 40 years old, which is close to the mean age of doctors, nurses and allied health professionals working in the Hospital Authority. Majority of our respondents were nurses and 72.8% of our respondents were females, which is similar to the nurse gender ratio working in the Hospital Authority. Therefore, respondents of our survey can be considered a representative sample.

Our study highlighted the key factors at the individual, family, and policy level affecting HCWs’ perceived level of stress and risk of being infected by COVID-19 for themselves and their family members during a pandemic. These factors included the nature of their work, change in family relationship during the outbreak, hospital policies, and prior experience of the SARS outbreak. Provision of adequate PPE and special allowance to the HCWs show great promise in protecting the psychological wellbeing of HCWs in fighting a pandemic.

## Data Availability

The raw data supporting the conclusions of this article will be made available by the authors, without undue reservation.
