# Supplementary material for: Multilevel Factors Affecting Healthcare Workers’ Perceived Stress and Risk of Infection During COVID-19 Pandemic
Source: Int J Public Health. 2021 Mar 5;66:599408. doi: 10.3389/ijph.2021.599408 (PMC8565288; doi:10.3389/ijph.2021.599408)
Supplement: Supplementary file 1 [file DataSheet1.PDF]

Impact of new coronavirus disease (COVID-19) on healthcare workers and their families (questionnaire_FM_Eng v1-20200303)

Chinese name of the healthcare worker who forwarded this survey link to you: ____________________

Mobile phone number of the healthcare worker who forwarded this survey link to you: _____________

Part A - Intrapersonal/individual factors

Demographics

1. You are:

○ Healthcare worker's spouse
○ Non-spouse family members (living with the healthcare worker)
○ Non-spouse family members (NOT living with the healthcare worker)
○ Others (please specify: ________________)

2. Your sex is:

○ Male ○ Female ○ Prefer not to say

3. Your age group is:

○ 18-24 ○ 25-34 ○ 35-44 ○ 45-54 ○ 55-64 ○ 65 or above ○ Prefer not to say

4. Have you been diagnosed with a psychiatric disorder or mood problem?

○ Yes
○ No
○ Prefer not to say

5. Do you have any chronic disease (e.g. hypertension, diabetes)?

○ Yes
○ No
○ Prefer not to say

6. Are you also working in a medical facility (including hospital and clinics)?

○ No
○ Yes, I am a ○ doctor ○ nurse ○ allied health professional ○ healthcare assistant ○ others, please specify: ______________________
○ Prefer not to say

7. Do you also work in the high-risk COVID-19 areas?

○ No
○ Yes
○ Prefer not to say

Perceived stress

The questions in this scale ask you about your feelings and thoughts **<u>during the COVID-19 outbreak</u>**.	**Never**	**Almost Never**	**Sometimes**	**Fairly Often**	**Very Often**
1. How often have you been upset because of something that happened unexpectedly?	☐$_0$	☐$_1$	☐$_2$	☐$_3$	☐$_4$
2. How often have you felt that you were unable to control the important things in your life?	☐$_0$	☐$_1$	☐$_2$	☐$_3$	☐$_4$
3. How often have you felt nervous and “stressed”?	☐$_0$	☐$_1$	☐$_2$	☐$_3$	☐$_4$
4. How often have you felt confident about your ability to handle your personal problems	☐$_0$	☐$_1$	☐$_2$	☐$_3$	☐$_4$
5. How often have you felt that things were going your way?	☐$_0$	☐$_1$	☐$_2$	☐$_3$	☐$_4$
6. How often have you found that you could not cope with all the things that you had to do?	☐$_0$	☐$_1$	☐$_2$	☐$_3$	☐$_4$
7. How often have you been able to control irritations in your life?	☐$_0$	☐$_1$	☐$_2$	☐$_3$	☐$_4$
8. How often have you felt that you were on top of things?	☐$_0$	☐$_1$	☐$_2$	☐$_3$	☐$_4$
9. How often have you been angered because of things that were outside of your control?	☐$_0$	☐$_1$	☐$_2$	☐$_3$	☐$_4$
10. How often have you felt difficulties were piling up so high that you could not overcome them?	☐$_0$	☐$_1$	☐$_2$	☐$_3$	☐$_4$

Family relationship

Please indicate how satisfied you felt towards your family **during the COVID-19 outbreak.**

	Hardly Ever	Some of the Time	Almost Always
1. I am satisfied that I can turn to my family for help when something is troubling me.	☐0	☐1	☐2
2. I am satisfied with the way my family talks over things with me and shares problems with me.	☐0	☐1	☐2
3. I am satisfied that my family accepts and supports my wishes to take on new activities or challenges.	☐0	☐1	☐2
4. I am satisfied with the way my family expresses affection and responds to my emotions, such as anger, sorrow and love.	☐0	☐1	☐2
5. I am satisfied with the way my family and I share time together.	☐0	☐1	☐2

6. Compared to your family relationship quality **before the COVID-19 outbreak**, how do you rate the **current** family relationship quality?

Much better	Better	Same	Worse	Much worse
☐0	☐1	☐2	☐3	☐3

Perceived Risk

How bad do you feel if **you** were diagnosed with the following diseases?

	(0) Not bad at all	(1) Not bad	(2) Neutral	(3) Bad	(4) Very bad
1. Seasonal influenza	☐	☐	☐	☐	☐
2. Cancer	☐	☐	☐	☐	☐
3. AIDS	☐	☐	☐	☐	☐
4. SARS in 2003	☐	☐	☐	☐	☐
5. Swine flu in 2009	☐	☐	☐	☐	☐
6. COVID-19	☐	☐	☐	☐	☐

How bad do you feel if **the healthcare worker who forwarded this survey to you** were diagnosed with the following diseases?

	(0) Not bad at all	(1) Not bad	(2) Neutral	(3) Bad	(4) Very bad
1. Seasonal influenza	☐	☐	☐	☐	☐
2. Cancer	☐	☐	☐	☐	☐
3. AIDS	☐	☐	☐	☐	☐
4. SARS in 2003	☐	☐	☐	☐	☐
5. Swine flu in 2009	☐	☐	☐	☐	☐
6. COVID-19	☐	☐	☐	☐	☐

Satisfaction with Special Measures for Hospital Staff

Please indicate the degree to which you **currently** agree or disagree with the following items.

	(0) Strongly Disagree	(1) Disagree	(2) Neutral	(3) Agree	(4) Strongly Agree
1. The protective gear for staff working in high-risk COVID-19 is sufficient.	☐	☐	☐	☐	☐
2. The protective gear for staff working in general wards is sufficient.	☐	☐	☐	☐	☐
3. The special accommodation allowance for staff working in high-risk COVID-19 areas is sufficient.	☐	☐	☐	☐	☐
4. The special cash allowance for staff working in high-risk COVID-19 areas is sufficient.	☐	☐	☐	☐	☐

Hopefulness for Hong Kong Future

1. Please indicate the extent to which you feel **worried about the COVID-19 epidemic in Hong Kong** on a scale of 0 = none at all to 100 = totally positive: _________________

1. Please indicate the extent to which you feel **hopeful about the future of Hong Kong** on a scale of 0 = none at all to 100 = totally positive: _________________

Follow-up Survey

After the COVID-19 outbreak, would you permit us to contact you to conduct a follow-up survey? Your participation will enhance our understanding of the conditions of family members of healthcare workers during the disease outbreak. Thank you so much!

○ No

○ Yes, name : ________________ contact phone (mobile phone is preferred): ___________________

Thank you for participation in this survey!
