# Supplementary material for: Multilevel Factors Affecting Healthcare Workers’ Perceived Stress and Risk of Infection During COVID-19 Pandemic
Source: Int J Public Health. 2021 Mar 5;66:599408. doi: 10.3389/ijph.2021.599408 (PMC8565288; doi:10.3389/ijph.2021.599408)
Supplement: Supplementary file 2 [file DataSheet2.PDF]

Impact of new coronavirus disease (COVID-19) on healthcare workers and their families (questionnaire_Eng v2-20200319)
Your Chinese name: ________________
Your mobile phone number: _________________

Part A - Intrapersonal/individual factors

Demographics

1. Your sex is:
○ Male ○ Female

2. Your age group is:
○ 18-24 ○ 25-34 ○ 35-44 ○ 45-54 ○ 55-64 ○ 65 or above ○ Prefer not to say

3. Your current marital status:
○ Single ○ Married/Cohabitation

4. I am a ○ doctor ○ nurse ○ allied health professional ○ healthcare assistant ○ others, please specify: _______________

5. Which hospital cluster are you currently serving?
○ Not applicable
○ Hong Kong East Cluster
○ Hong Kong West Cluster
○ Kowloon Central Cluster
○ Kowloon East Cluster
○ Kowloon West Cluster
○ New Territories East Cluster
○ New Territories West Cluster
○ Prefer not to say

6. Your duty during the COVID-19 epidemic:
○ Currently working in Isolation Unit ○ Was working in Isolation unit
○ Will be working in Isolation unit ○ Working in Fever ward in non-isolated ward
○ Working in Emergency Department ○ Working in General out-patient clinic
○ Working in specialty without isolation unit, please specify:_____________

7. Did you temporarily live in a separate location (such as hotel or hospital accommodation) because of the current COVID-19 outbreak?
○ Yes ○ No

8. Has you had direct contact with the COVID-19 confirmed case:
○ Yes ○ No

9. During the atypical pneumonia (SARS) outbreak in 2003, did you experience the outbreak in Hong Kong?
○ Yes ○ No

10. During the atypical pneumonia (SARS) outbreak in 2003, did you work in the healthcare sector?
○ Yes ○ No

11. During the atypical pneumonia (SARS) outbreak in 2003, did you work as front-line workers to take care of patients who were infected at the time?
○ Yes ○ No

12. During the atypical pneumonia (SARS) outbreak in 2003, did any of your peers / colleagues / relatives become infected or die as a result of the infection?
○ Yes ○ No

13. How many years have you worked in the healthcare industry:
○ 1-3 ○ 4-6 ○ 7-9 ○ 10-12 ○ 13-15 ○ 15-17 ○ 18-20 ○ 20 or above

14. How many children do you have: ____________

15. How many children are currently living with you: ____________

16. Are you living with elders:
○ Yes ○ No

17. Have you been diagnosed with a psychiatric disorder or mood problem?
○ Yes ○ No

18. Do you have any chronic disease (e.g. hypertension, diabetes)?
○ Yes ○ No

Perceived stress

The questions in this scale ask you about your feelings and thoughts **during the COVID-19 outbreak**.	**Never**	**Almost Never**	**Sometimes**	**Fairly Often**	**Very Often**
1. How often have you been upset because of something that happened unexpectedly?	☐0	☐1	☐2	☐3	☐4
2. How often have you felt that you were unable to control the important things in your life?	☐0	☐1	☐2	☐3	☐4
3. How often have you felt nervous and "stressed"?	☐0	☐1	☐2	☐3	☐4
4. How often have you felt confident about your ability to handle your personal problems	☐0	☐1	☐2	☐3	☐4
5. How often have you felt that things were going your way?	☐0	☐1	☐2	☐3	☐4
6. How often have you found that you could not cope with all the things that you had to do?	☐0	☐1	☐2	☐3	☐4
7. How often have you been able to control irritations in your life?	☐0	☐1	☐2	☐3	☐4
8. How often have you felt that you were on top of things?	☐0	☐1	☐2	☐3	☐4
9. How often have you been angered because of things that were outside of your control?	☐0	☐1	☐2	☐3	☐4
10. How often have you felt difficulties were piling up so high that you could not overcome them?	☐0	☐1	☐2	☐3	☐4

Family relationship

Please indicate how satisfied you felt towards your family **during the COVID-19 outbreak.**

	**Hardly Ever**	**Some of the Time**	**Almost Always**
1. I am satisfied that I can turn to my family for help when something is troubling me.	☐0	☐1	☐2
2. I am satisfied with the way my family talks over things with me and shares problems with me.	☐0	☐1	☐2
3. I am satisfied that my family accepts and supports my wishes to take on new activities or challenges	☐0	☐1	☐2
4. I am satisfied with the way my family expresses affection and responds to my emotions, such as anger, sorrow and love.	☐0	☐1	☐2
5. I am satisfied with the way my family and I share time together.	☐0	☐1	☐2

6. Compared to your family relationship quality **before the COVID-19 outbreak**, how do you rate the **current** family relationship quality?

Much better	Better	Same	Worse	Much worse
☐0	☐1	☐2	☐3	☐3

Perceived Risk

How bad do you feel if **you were diagnosed** with the following diseases?

	(0) Not bad at all	(1) Not bad	(2) Neutral	(3) Bad	(4) Very bad
1. COVID-19	☐	☐	☐	☐	☐
2. Seasonal influenza	☐	☐	☐	☐	☐
3. Cancer	☐	☐	☐	☐	☐
4. SARS in 2003	☐	☐	☐	☐	☐
5. Swine flu in 2009	☐	☐	☐	☐	☐

How bad do you feel if **your family members were diagnosed** with the following diseases?

	(0) Not bad at all	(1) Not bad	(2) Neutral	(3) Bad	(4) Very bad
1. COVID-19	☐	☐	☐	☐	☐
2. Seasonal influenza	☐	☐	☐	☐	☐
3. Cancer	☐	☐	☐	☐	☐
4. SARS in 2003	☐	☐	☐	☐	☐
5. Swine flu in 2009	☐	☐	☐	☐	☐

Satisfaction with Special Measures for Hospital Staff

Please indicate the degree to which you **currently** agree or disagree with the following items.

	(0) Strongly Disagree	(1) Disagree	(2) Neutral	(3) Agree	(4) Strongly Agree
1. The protective gear for staff working in high-risk COVID-19 is sufficient.	☐	☐	☐	☐	☐
2. The protective gear for staff working in general wards is sufficient.	☐	☐	☐	☐	☐
3. The special accommodation allowance for staff working in high-risk COVID-19 areas is sufficient.	☐	☐	☐	☐	☐
4. The special cash allowance for staff working in high-risk COVID-19 areas is sufficient.	☐	☐	☐	☐	☐

Hopefulness for Hong Kong Future

1. Please indicate the extent to which you feel **worried about the COVID-19 epidemic in Hong Kong** on a scale of 0 = none at all to 100 = totally positive: ________________

2. Please indicate the extent to which you feel **hopeful about the future of Hong Kong** on a scale of 0 = none at all to 100 = totally positive: ________________

Follow-up Survey

After the COVID-19 outbreak, would you permit us to contact you to conduct a follow-up survey? Your participation will enhance our understanding of the conditions of healthcare workers during the disease outbreak. Thank you so much!

○ Yes

○ No

Gift

Thank you for completing the survey! To show our appreciation for your participation in this survey, we prepared a HKD 100 gift (basic infection control kit).

Disclaimer:

1. Each family (i.e. the healthcare worker respondent and his/her family) can only receive one kit.
2. Please come to the research team's office in Queen Mary Hospital to pick up the gift.
3. If the phone number you provided in this survey were also recorded in other healthcare workers' survey responses, our research team would call you to verify and discuss the subsequent gift arrangement.
4. This limited-number offer is only available while stock lasts.
5. The University of Hong Kong has the right to make the final decision should any dispute arise.
6. For enquiry, please contact the research team at 2819 8065.

Do you want to have the gift?

○ No need (we would donate it to the needy ones on your behalf)

○ I want to have adult-sized masks and one bottle of 50ml alcohol hand-rub and agree on the above arrangement.

○ I want to have child-sized masks and one bottle of 50ml alcohol hand-rub and agree on the above arrangement.

Thank you for participation in this survey! Please forward the family member survey link to your family member!
